# Three Cases of Excision of the Hip-Joint

**Published:** 1883-10

**Authors:** John E. Owens

**Affiliations:** Prof. of Surg. Anat. and Operative Surgery, Chicago Medical College; No. 112 E. Monroe Street


### ARTICLE V.

THREE CASES OF EXCISION OF THE HIP JOINT. By Jno. E. Owens, M.D., Prof. of Surg. Anat. and Operative Surgery, Chicago Medical College. Reported by Frank L. Rea, M.D., late Interne St. Luke's Hospital.

In the spring of 1882, Prof. Owens gave a course of surgical clinics in St. Luke's Hospital to the gentlemen of the practitioners' course of the Chicago Medical College. At each of the first three of these regular Monday afternoon clinics, which are continued through the college year, he excised the hip joint for hip disease. The patients were all natives of Illinois, one living in this city and the two others in the country. Neither had ever enjoyed but fair general health. The patients were, S—— K——, a female aged ten years, an orphan, with a moderately marked phthisical hereditary history. She had been suffering from hip-joint disease two and one-half years, all treatment having been neglected.

F—— Y——; was six years of age, and in addition to the hip disease had Pott's disease high in the dorsal region. His father was subject to attacks of hæmoptysis; mother healthy, and sister myopic and more or less strumous. This case seemed to present such difficulties of diagnosis that the hip disease was not made out with certainty until the joint suppurated. It was recognized when he applied for admission to the hospital, and the abscess communicating with the joint was opened. Up to the entrance into the institution it had progressed six months. The disease was not recognized in this case at a time when conservative measures were of utility.

L—— K——; of healthy Irish parentage—the youngest of a large family of healthy children, was aged five years. He had been suffering from hip disease for two (2) years. Orthopædic measures had been given an early and thorough trial by competent hands in this boy's case, but effected nothing. No assigned cause, as injury, was given for either case. Prof. Owens shows that many cases of hip disease and Pott's disease are of traumatic origin. He also affirms that the scrofulous appearance of this class of patients is in very many cases due to the bone disease, and that it does not by any means always bear a causative relation to it. The diseased hip-joint in each was disorganized, and the hip riddled with sinuses discharging pus in enormous quantities. All presented the pale transparent skin, fair hair, blue eyes, glandular enlargements, small muscles, large joints, tissues weak and of flabby texture, large abdomens, precocious minds, etc. The affected limb was adducted and shortened, the flexion of the thigh on the pelvis causing marked lordosis.

The muscular atrophy from want of use was great in the gluteal region, but existed in the whole limb when compared with the other one. The children were, in this stage of the disease, only occasionally affected with pain of any consequence. Dead bone was made out with the probe in each case in the situation of the head of the femur. Preliminary to the operation, which was demanded by the necessities of each patient, they were put upon quinine and iron, which, with the improved hygiene of the institution over that of their homes, effected such improvement in their general health as rendered them fair subjects for operation in about one month.

*Operation:*—Being the same in the three cases, one description serves for all. During the administration of the ether Prof. Owens gave as the indications for the operation: (1) Failure of orthopædic measures; (2) extensive bone mischief, with copious suppuration; (3) bad general condition; (4) in his experience the operation has been a very successful one in childhood; and again, he remarked, what does further conservatism promise? death from exhaustion or amyloid degeneration of the viscera, or a remote and tedious recovery by anchylosis of the diseased joint. Patient being anæsthetized, and the part to be operated upon carefully cleansed and carbolized, Prof. Owens proceeded to operate, first verifying the presence of the dead bone with the probe, then with a strong scalpel beginning the curved incision, concave forwards from two inches above the greater trochanter to two inches below it, along its posterior border down to the bone. This was now denuded of its periosteum and soft parts by the elevator, and what remained of the carious articular surfaces was found in opposition. In regard to this point, Prof. Owens admits deformity resembling luxation, but the cause was always caries with absorption of bone, and in his now rather large experience he has never found the surfaces other than as in these cases, and is skeptical regarding the occurrence of the so-called spontaneous dislocation. The lips of the incision were now held apart by retractors, and the bone sawn through its upper extremity above the lesser trochanter with an Adams' saw; not, however, without previously attempting to turn the head of the bone out of its socket. This maneuver failed, as it occasionally does, in consequence of an involucrum formed around the remains of the joint by long-continued inflammation. What remained of the head, neck and greater trochanter were evulsed by the lion-jawed forceps.

Why is the greater trochanter removed when not involved in the caries? Because if left (1) it serves as a constant irritant to the tissues. (2) It is next to impossible to keep it away from the pelvic bones, causing pain from friction of the roughened bony surfaces. (3) It forms the outer wall of a pocket which cannot be efficiently drained. The last is the chief cause for its removal. A considerable extent of carious bone in the acetabulum in one case, L——. K———., was removed with the bone scraper. All of the sinuses which had led into the diseased joint through the undermined tissues, were slit up into the excision wound upon a grooved director. The condition of these sinuses, many of them containing particles of carious bone, made this the only means of securing the necessary cleanliness and drainage to insure their healing from the bottom. The hæmorrhage was slight, and in no instance did a vessel require ligation. The operation lasted thirty minutes. The wound was now irrigated with a solution of carbolic acid 1-40, sponged dry, poured full of balsam of Peru, and completely filled by a tent made of a rope of oakum. The whole hip was now covered with a compress of oakum, and all retained by a bandage. The patient was then put into a Sayre's wire cuirass, the efficient padding of which is so important that it should receive the physician's personal attention. It is done in this way: The wire splint is covered with sheet wadding, cut in the proper shape and in sufficient quantity to insure thickness and softness. These layers are then covered with a rubber cloth, which is sewed on, smoothness being very essential. If this is not followed out, it will require more than ordinary care to prevent bed-sores. In addition to the permanent padding, a number of layers of sheet wadding were always put between the child and the splint. The extension of the shortened limb was effected by means of adhesive straps passing from the sides of the leg over the foot-piece, which was adjusted by means of a screw. The foot of the sound leg resting against its foot-piece furnished the point of support or counter extension. The body and both lower limbs were neatly bandaged to the splint. Sufficient extension only was kept up to prevent friction of the roughened bony surfaces, thereby preventing much pain. A practical point in this connection is that pain in the knee and the hip was often due to this cause, and received prompt relief by a few turns of the screw slightly increasing the extension. An anodyne was administered on recovery from the ether, and subsequently repeated when pain or nervousness called for it. Shock from the operation was slight.

Surgical fever in but one case went above 102°, lasting a few days and falling to normal. The dressings were removed forty-eight hours after the operation; the wound irrigated with a 1-40 solution of carbolic acid, thoroughly repacked with oakum saturated with balsam of Peru, and the whole hip covered with a compress of oakum placed between two layers of gauze—the inner one being lubricated with vaseline to facilitate its removal. From this time up to the discharge of the patients this was done once daily, a tent, such as just described, being used until rendered unnecessary by granulation of the bottom of the wound. To protect the splint and the bed from the discharges of the patient, early teach the child to make his wants known, when by a suitable vessel and care in the position of the splint, the dressings are kept clean. Once weekly the cuirass was cleansed, the patient being removed for the purpose. The cuirass was omitted entirely after a time, averaging two months for each case. It is done gradually, thus: leaving it off for a few hours every other day at first, increasing time and interval so that in a short time it is laid away. In about two weeks after effecting this, apparatuses were put on each case.

In the case complicated with Pott's disease, F—— Y——, the apparatus consisted of a plaster jacket, a pair of crutches, and for the foot of the short limb a shoe, the sole of which was augmented by four ounces of lead. This limb being sufficiently short, no elevation of the sound side was required. He was from this time on taught, gradually and carefully, to walk, and left the hospital with the apparatus on four months after the operation. Six months later the apparatus, with the exception of the crutches, was discarded, the case illustrating beautifully a cure of a Pott's disease and of a hip disease with a movable joint.

In the case of S—— K——, a plaster jacket was also put on, and to this was attached, by a few turns of the plaster roller, an iron frame which supported a ball and socket joint opposite that of the hip. From this a steel bar, with ratchet in thigh piece, extended down the outer side of the limb, fastening to the sole of the shoe below; another bar extended along the inner side of the limb to the upper part of the thigh. These bars had hinge joints at knee and ankle, the former being a lock joint. They were held to the limb by leather bands buckling in front. Extension was effected in this case by means of the ratchet in the external thigh bar. This patient was also discharged about four months after the operation, with the apparatus still in use. A movable joint was secured in this case.

L—— K—— had applied a splint consisting of a leather pelvic band with a steel bar extending down the outer side of the limb to the sole of the shoe, and another one from this place along the inner side of the limb to the upper part of the thigh. These bars had hinge joints at the knee and the ankle, and the external thigh bar one opposite the hip-joint. This splint was put on to correct the marked internal rotation of the limb that had taken place. Extension was effected by a weighted shoe as above described. A pair of crutches completed the apparatus. The boy was taught to walk, and discharged in good condition, with a movable joint, seven months after the operation. The convalescence was protracted from leaving the tent out too soon, the wound healing superficially and requiring a secondary operation to secure drainage and make it heal from the bottom. In this operation the upper end of the femur was exposed and found to be smooth and rounded. This case has since (May, 1883), returned to the hospital with three sinuses leading down to carious bone. Considerable pus was being discharged. His general condition was much worse than when he left the institution. Ether was administered, and an incision two inches long was made in the line of the old cicatrix. Carious bone was found in the acetabulum and removed with the bone-scraper. A drainage tube was inserted and brought out at an opening two inches lower down. The wound was packed with lint and a gauze dressing put on.

An interesting fact in connection with these cases was the early and marked improvement in the general health which followed the removal of the carious bone.

July 20, 1883.

L—— K——— is in the hospital, convalescing favorably from the last operation. He is up and about, using apparatus consisting only of a pair of crutches and a weighted shoe for foot of short limb.

F—— Y——— is at home; all of the the sinuses healed; using no apparatus of any character.

Report from S—— K——— at this date is not favorable. The sinuses not healed are discharging pus, and doubtless lead to carious bone. The new joint is almost immovable. She is up and about, assisted by a crutch. She eats and sleeps well. This child was unfortunate in being removed to quarters where she did not receive the requisite surgical supervision. Her plaster jacket was left on for a year; passive motion of the new joint was certainly neglected, as shown by the anchylosis which took place after she was taken away from St. Luke's; and further, I infer from the report received that she has been seen but two or three times by a physician during this time. This case illustrates that patients of this kind require the watchful eye of the surgeon until full recovery has taken place.

Prof. Owens entertains the following views on this subject:

1. He recommends removal of the greater trochanter in the excision.

2. He asserts that traumatism, not scrofula, is the cause of the disease in a majority of cases.

3. He is skeptical regarding the spontaneous dislocation of the third stage of hip-disease.

4. In the treatment after excision, he places much value on the employment of an apparatus with a ball and socket joint opposite that of the hip, in effecting a cure with a movable joint.

5. With additional experience he becomes a stronger advocate of early excision.

No. 112 E. MONROE STREET.

